# The association between body composition and metabolically unhealthy profile of adults with normal weight in Northwest China

**DOI:** 10.1371/journal.pone.0248782

**Published:** 2021-03-25

**Authors:** Ling Fan, Jiangwei Qiu, Yu Zhao, Ting Yin, Xiaoxia Li, Qingan Wang, Jinyun Jing, Jiaxing Zhang, Faxuan Wang, Xiuying Liu, Lan Liu, Yi Zhao, Yuhong Zhang

**Affiliations:** 1 School of Public Health and Management, Ningxia Medical University, Yinchuan, Ningxia, China; 2 The Key Laboratory of Environmental Factors and Chronic Disease Control of Ningxia, Yinchuan, Ningxia, China; Medical University of Vienna, AUSTRIA

## Abstract

**Objective:**

Related evidences of metabolically unhealthy profile of adults with normal weight are not well characterized in the Chinese population. This is because they cannot be effectively identified by regular measurements (such as body mass index [BMI]). To overcome this gap in literature, this study aimed at investigating the association between body composition and metabolically unhealthy profile in Chinese adults with normal weight.

**Methods:**

A total of 5427 individuals with normal-weight were recruited from 15820 people living in Ningxia Hui Autonomous Region in Northwest China. Normal-weight was defined as a BMI of 18.5–23.9 kg/m^2^. Metabolically unhealthy profile was assessed by the National Cholesterol Education Program Adult Treatment Panel III (ATP III). Metabolically unhealthy normal-weight (MUHNW) profile was defined in individuals who had normal weight and at least two cardiometabolic risk factors. Generalized linear model was used to investigate the association between body composition measured by bioelectrical impedance and metabolically unhealthy profile in adults with normal-weight.

**Results:**

The percentage of metabolically unhealthy profile was 35.86% in adults with normal weight. Different MUHNW distributions were found between males and females depending on age. The percentage of the MUHNW profile significantly increased in women after the age of 55, contrary to men. The association between body composition and MUHNW was affected by age and sex. The increased adiposity indices (fat mass index [FMI], visceral fat level [VFL], waist circumference [WCF]), and reduced skeletal muscle mass ratio [SMR] showed significant differences between MUHNW and metabolically healthy with normal weight (MHNW) (p < 0.05).

**Conclusion:**

The distribution of MUHNW differed between ages and sexes. FMI, VFL, WCF and SMR could be responsible for the MUHNW adults, providing a new insight into the potential metabolic risks for the adults with normal weight in China. This directs us in the management of the MUHNW for their early prevention.

## 1. Introduction

With the development of economies, high-nutrient diets have caused a considerable increase of the prevalence of obesity worldwide. According to the latest data published by the World Health Organization, the prevalence of overweight and obesity among adults are 39% and 13%, respectively [1]. There is quite some heterogeneity in the relationship between obesity and risk of cardiometabolic diseases, and some obese individuals have a relatively low risk of cardiometabolic diseases [2]. However, most obese individuals have an increased risk for cardiometabolic diseases, such as hypertension, dyslipidemia, diabetes, and cardiovascular disease. Thus, the increased obesity imposes a heavy social burden and has gained public health concerns recently [3].

As already known, body mass index (BMI) is viewed as a diagnostic measurement for obesity. Individuals in the normal BMI range may also display metabolically unhealthy profile associated with obesity [4]. Ruderman et *al*. [5] summarized that individuals with normal weight (or BMI), but like people with obesity, have hyperinsulinemia, insulin-resistance, and predisposed to type 2 diabetes, hypertriglyceridemia, and premature coronary heart disease. Generally, it is difficult to identify the individuals with normal weight who are metabolically unhealthy by using BMI measurements. In fact, some individuals with normal weight have a metabolically unhealthy profile worldwide. Specifically, Asians have higher percent body fat with a lower BMI [6]. Recently, the metabolically unhealthy normal-weight (MUHNW) phenomenon was reported widespread and serious in Asian countries.

The latest results showed that individuals with MUHNW may be predisposed to similar adverse health outcomes as those observed with obesity. The cardiovascular disease risk of MUHNW was considerably higher than that of metabolically healthy with normal weight (MHNW) and MHO (metabolically healthy obesity) in a prospective cohort study follow-up of 30 years [7]. A prospective cohort study with a median follow-up of ten years showed that older individuals with MUHNW exhibited greater all-cause mortality than MHO [8]. A systematic review and meta-analysis of prospective studies suggests that the risk of cardiovascular disease events was higher for MUHNW than MHO [9].

However, since the individuals with MUHNW have normal-weight, they may not attract adequate personal or medical attention, which will increase the risk of untreated complications. Therefore, identifying the potential risk factors of metabolically unhealthy profile for the individuals with normal weight in China plays an important role in formulating early intervention strategies.

Moreover, the existing guidelines also failed to individualize the management of the individuals with MUHNW. There still exists a gap between the understanding and appropriate management of individuals with normal weight, with a probably high risk of metabolically unhealthy profile. Accurate classification and mechanistic understanding for these individuals can provide an optimal health care and appropriate early treatment as well as decrease health care costs due to improper treatments and requisite subsequent medical interventions [10].

Human body composition (BC) has been extensively measured in individuals with normal weight and obesity. An analysis found that [11] lean people unexpectedly have a risk of type 2 diabetes and cardiovascular disease that is similar to the increased risk that is observed in most individuals with obesity. Not only visceral fat mass has strong predictive power of the risk of type 2 diabetes and cardiometabolic disease [12], but also an impaired ability to expand subcutaneous fat in the lower part of the body. This implied that BC plays an important role in predicting the risk of cardiometabolic diseases of individuals with normal weight or obesity. Although BMI has been a widely used index to evaluate normal weight and obesity, it has limitations in differentiating body fat from lean mass [13]. Thus, we sought to evaluate whether the use BC to replace BMI as an easily detectable and cost-effective measurement for identifying the metabolically unhealthy profile in individuals with normal weight is a meaningful issue.

The percentage of MUHNW showed the different distributions between male and female. However, the research results on this respect are still not consistent. For instance, Eckel et *al*. [14] found that the male sex is one of the MUHNW risk factor, but Zhang et *al*. [15] showed that the female had a higher MUHNW percentage. There are also some reported results that focused on the Caucasians population using MR-based measurements [16]. However, the related evidence based on the Chinese or Asian populations was still limited. Therefore, the objective of the study is to assess the association between BC and metabolically unhealthy profile in adults with normal weight in rural China.

## 2. Materials and methods

### 2.1 Study participants and recruitment design

**Fig 1:**
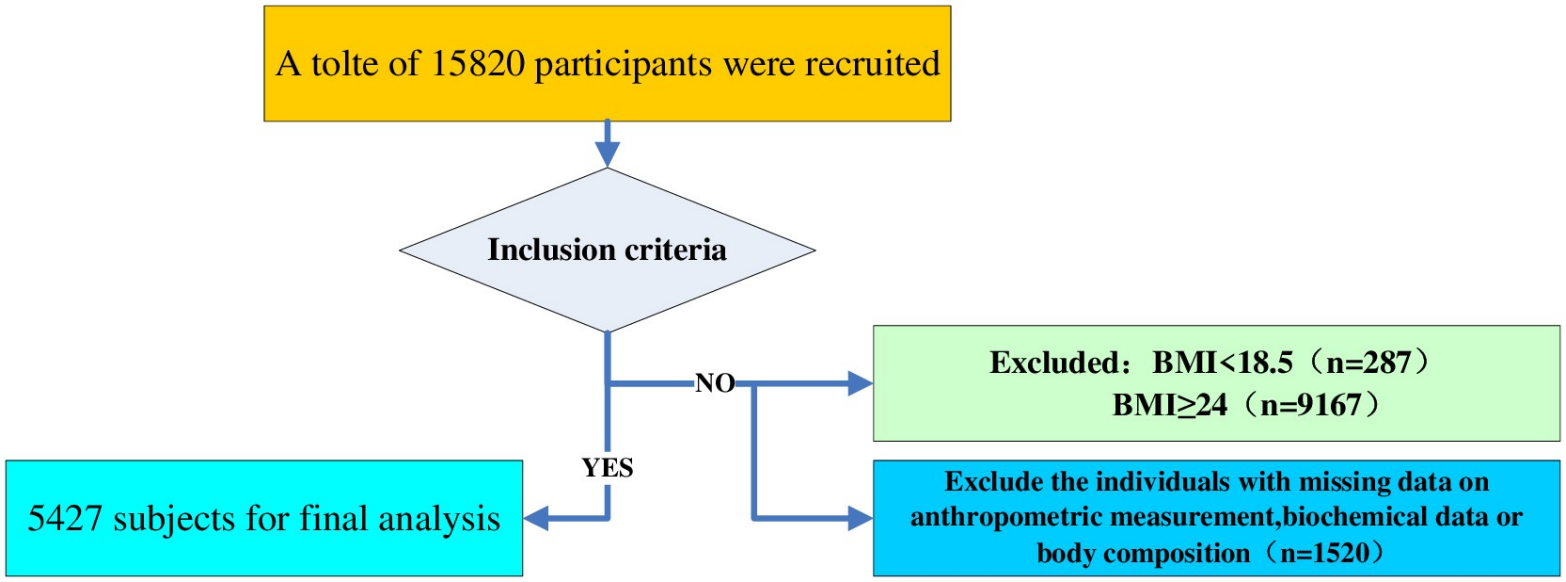
Flows chart of the study. 5427 individuals were recruited from 15820 individuals in the CNC-NX cohort.

The participants were recruited from the baseline database of the China North-West Natural Population Cohort: Ningxia Project (CNC-NX). The CNC-NX cohort is a prospective study regarding dietary patterns on non-communicable diseases. The baseline investigation was a community cluster study for the rural population in Ningxia Hui Autonomous Region, located in North west China. Two communities (referred to as ‘townships’) in each county, which county was selected randomly in Wuzhong and Shizuishan city, were selected by the local Centre for Diseases Control and Prevention in consideration of the coherence of residents, population stability and local economical and medical conditions. The participants were recruited through health examination in the public health institutions (such as community health service stations). We enrolled 15,820 participants into the CNC-NX cohort, and the baseline investigations were carried out in 2018 and 2019, respectively. In this work, we mainly focus on the baseline investigation, and a recruitment flow chart of participants was displayed as in Fig 1. In addition, participants with acute disease such as acute myocardial infarction/stroke, severe asthma/COPD, inflammatory bowel disease, and cancer were excluded.

The study was approved by the Ningxia Medical University Ethics Committee, and all participants obtained written and verbal information about our study and gave written informed consent. The study methods included using face-to-face questionnaires, physical measurements, and fasting blood collections.

### 2.2 Anthropometric and biochemical variables

#### 2.2.1 Face-to-face questionnaire survey

Demographic information was obtained through interview with structured questionnaire. Parameters obtained include: sex, education level, smoking status, alcohol drinking status, tea drinking status, physical exercise, and sleep disorders (Table 1). The alcohol drinking status was divided into two categories: alcohol drinkers (yes) and alcohol nondrinkers (no) according to whether they had a weekly drinking experience lasting at least a year or not.

**Table 1:** Baseline characteristics of participants stratified by BMI and metabolic status.

Variables	MHNW (n = 3481)	MUHNW (n = 1946)	χ^2^/t
Demography/anthropometry			
Age (years)	56.01 ± 10.24	58.62 ± 9.85	9.11*
Sex, n (%)			41.93*
Male	1502 (43.1%)	665 (34.2%)	
Female	1979 (56.9%)	1281 (65.8%)	
Height (cm)	160.43 ± 7.82	159.80 ± 7.85	2.87*
Weight (kg)	56.17 ± 6.47	56.76 ± 6.45	3.25*
BMI (kg/m^2^)	21.78 ± 1.43	22.18 ± 1.31	10.50*
Education level			32.06*
Primary school or below	2216 (63.7)	1382 (71.0)	
Secondary school	1237 (35.5)	557 (28.6)	
College or upper	28 (0.8)	7 (0.4)	
Smoking status			21.06*
Non-smoking	2884 (82.8)	1701(87.4)	
Occasional smoking	141 (4.1)	63 (3.2)	
Smoking on most days	17 (0.5)	10 (0.5)	
Smoking everyday	439 (12.6)	172 (8.8)	
Alcohol drinking status			0.30
No	3394 (97.5)	1902 (97.7)	
Yes	87 (2.5)	44 (2.3)	
Tea drinking status			0.13
No	3329 (95.6)	1865 (95.8)	
Yes	152 (4.4)	81 (4.2)	
Physical exercise			5.29
Never or almost never	1395 (40.1)	817 (42.0)	
< 3 times/week	1034 (29.7)	521 (26.8)	
≥ 3 times/week	1052 (30.2)	608 (31.2)	
Sleep disorder			7.65*
No	2536 (72.9)	1349 (69.3)	
Yes	945 (27.1)	597 (30.7)	
Cardiometabolic risk			
SBP (mmHg)	126.64 ± 17.85	139.39 ± 18.28	25.02*
DBP (mmHg)	77.90 ± 11.44	84.52 ± 11.95	19.87*
FPG (mmol/L)	5.24 ± 1.32	6.29 ± 2.62	16.50*
TC (mmol/L)	4.71 ± 0.88	4.83 ± 1.10	4.19*
TG (mmol/L)	1.06, IQR (0.81,1.38)	1.79, IQR (1.27,2.38)	35.43*
HDL-C (mmol/L)	1.47 ± 0.41	1.23 ± 0.31	21.53*
LDL-C (mmol/L)	2.71 ± 0.81	2.87 ± 0.90	6.28*
Body composition			
FMI (kg/m^2^)	5.76 ± 1.55	6.31 ± 1.55	12.59*
VFL	6.43 ± 2.11	7.28 ± 2.28	13.47*
WCF (cm)	78.62 ± 5.23	80.23 ± 5.26	10.85*
SMR (%)	39.86 ± 3.90	38.65 ± 3.89	11.03*

*P<0.05. Continuous variables shown as mean ± SD.

#### 2.2.2 Clinical assessment

Height and weight were measured with the shoe of individuals off and with light clothing in an upright position on the height of the scale after calibration. The whole-body fat mass, visceral fat level (VFL), waist circumference (WCF) and skeletal muscle mass were respectively measured by multifrequency bioelectric impedance analysis (BIA, measured by bio-electrical impedance devices, InBody 370 system). According to the measures, the fat mass index (FMI, whole body fat mass divided by height squared) and the skeletal muscle mass ratio (SMR, skeletal muscle mass divided by the body weight) were subsequently calculated.

The BMI (weight in kilograms divided by height in meters squared) was calculated for each participant, and the cut-offs in adults recommended by the Working Group on Obesity in China were used to classify normal weight (18.5–23.9 kg/m^2^), overweight (24.0–27.9 kg/m^2^), and obesity (≥ 28 kg/m^2^) [17].

Electronic sphygmomanometers (OMRON HEM-801 model) were used to measure resting blood pressure and heart rate twice with the quality control. To ensure accurate recording of biophysical measurements, the participants were required to rest at least 5 mins, and tea, alcohol consumption, cigarette smoking, and excessive physical activity were forbidden before the measure at least 30 mins or longer.

#### 2.2.3 Fasting blood samples and laboratory tests

Venous blood samples were collected by direct venipuncture after overnight fasting. The samples were centrifuged, aliquoted, and immediately frozen for future analysis. Biochemical biomarkers analyzed included total cholesterol (TC), triglyceride (TG), high-density lipoprotein cholesterol (HDL-C) and low-density lipoprotein cholesterol (LDL-C) detected using biochemical auto-analyzers (Mindray BS-430, China).

### 2.3 Metabolically unhealthy normal weight profile definition

To data, there were some types of general identification criteria of metabolic syndrome (MetS) worldwide. For example, International Diabetes Federation (IDF) definition and Adult Treat panel (ATP) III definition, etc. Among these criteria of metabolic syndrome, systolic/diastolic blood pressure (SBP/DBP)/antihypertension medication use (AMU), fasting triglyceride (TG) level, fasting plasma glucose (FPG), waist circumference (WCF) and fasting HDL-cholesterol (HDL-C) level are the common traits. The main difference between ATP III and IDF definitions is that some cutoff value were different (TG, HDL-C), and IDF more restrict (FPG, WCF) [18]. A recently results shown that WCF may exist collinearity with BMI, thus, the simultaneous use of BMI and WCF was not recommended [19]. In IDF definition, the SBP/DBP and AMU are alternative criteria, TG or HDL-C and lipid lowering medication are alternative criteria, FPG or medication for diabetes mellitus are alternative criteria, however, only medication for diabetes mellitus and treatment of diagnosed hypertension were considered in ATP III as alternative criteria. In addition, in IDF definition, central obesity is a mandatory criterion for identifying MetS [20]. In our study, we focus on the metabolic unhealthy individuals with normal weight, the IDF definition may not suitable to identify such situation. Thus, based on above discussion, we selected a criterion based on ATP III that metabolically unhealthy normal-weight (MUHNW) profile was defined in individuals who had normal weight and at least two cardiometabolic risk factors. Therefore, we used the criteria established by the National Cholesterol Education Program Adult Treatment Panel III (ATP III) to identify the metabolic status [21]. The presence of at least two of the following items was defined as metabolically unhealthy profile: (1) high blood pressure (systolic blood pressure ≥ 130 or diastolic blood pressure ≥ 85 mmHg or known treatment for hypertension), (2) hypertriglyceridemia (fasting plasma triglycerides ≥ 1.7 mmol/L), (3) low HDL cholesterol (< 1.29 mmol/L for female or < 1.03 mmol/L for male) and (4) hyperglycemia (fasting plasma glucose ≥ 6.1 mmol/L or known treatment for diabetes). All the participants were divided into two groups: MHNW and MUHNW.

### 2.4 Statistical analysis

Data was analyzed using the SPSS software version 23.0 (IBM Corp., Armonk, New York) and R software (version 4.0.0). The R software was used to fill in the missing data (education level, smoking status, alcohol drinking status, tea drinking status, physical exercise, sleep disorder) using the “randomforest” package. The SPSS software was used for the rest of the analysis. Continuous variables were described by mean ($\bar{x}$) ± standard deviation (SD) and number (percentage, %) for categorical variables. Independent t-test or ANOVA (analysis of variance) was used to compare continuous variables between different groups. To compare the distribution of MUHNW in different groups, χ^2^ test was used. Generalized linear model (GLM) was conducted to examine the association between BC and metabolically unhealthy profile. Odds ratios (OR) and their 95% confidence intervals were calculated as well. All the tests were two-sided, and a value of p < 0.05 was considered as statistically significant.

## 3. Results

### 3.1 Baseline characteristics

We included 5427 participants (accounted for 34.3% in CNC-NX Cohort) with complete data on anthropometric measurements, blood indicators, and BC in our study. The average age of the population is 56.95 years old (ranged from aged 35 to aged 74 years).

The percentage of MUHNW was 35.86%, which was slightly higher than other related results [22]. The percentage of MUHNW was significantly different between males and females. The metabolically unhealthy individuals were more likely to be older. The cardiometabolic risk factors (SBP, DBP, FPG, TC, TG, LDL-C) were significantly lower in the MHNW group than in the MUHNW group. The FMI (*t* = 12.59, *P*<0.001), VFL (*t* = 13.47, *P*<0.001), and WCF (*t* = 10.85, *P*<0.001) in the MUHNW group were significantly higher than those in the MHNW group, but the SMR (*t* = 11.03, *P*<0.001) was significantly lower in the MUHNW group (Table 1).

### 3.2 Characteristics of body composition

**Fig 2:**
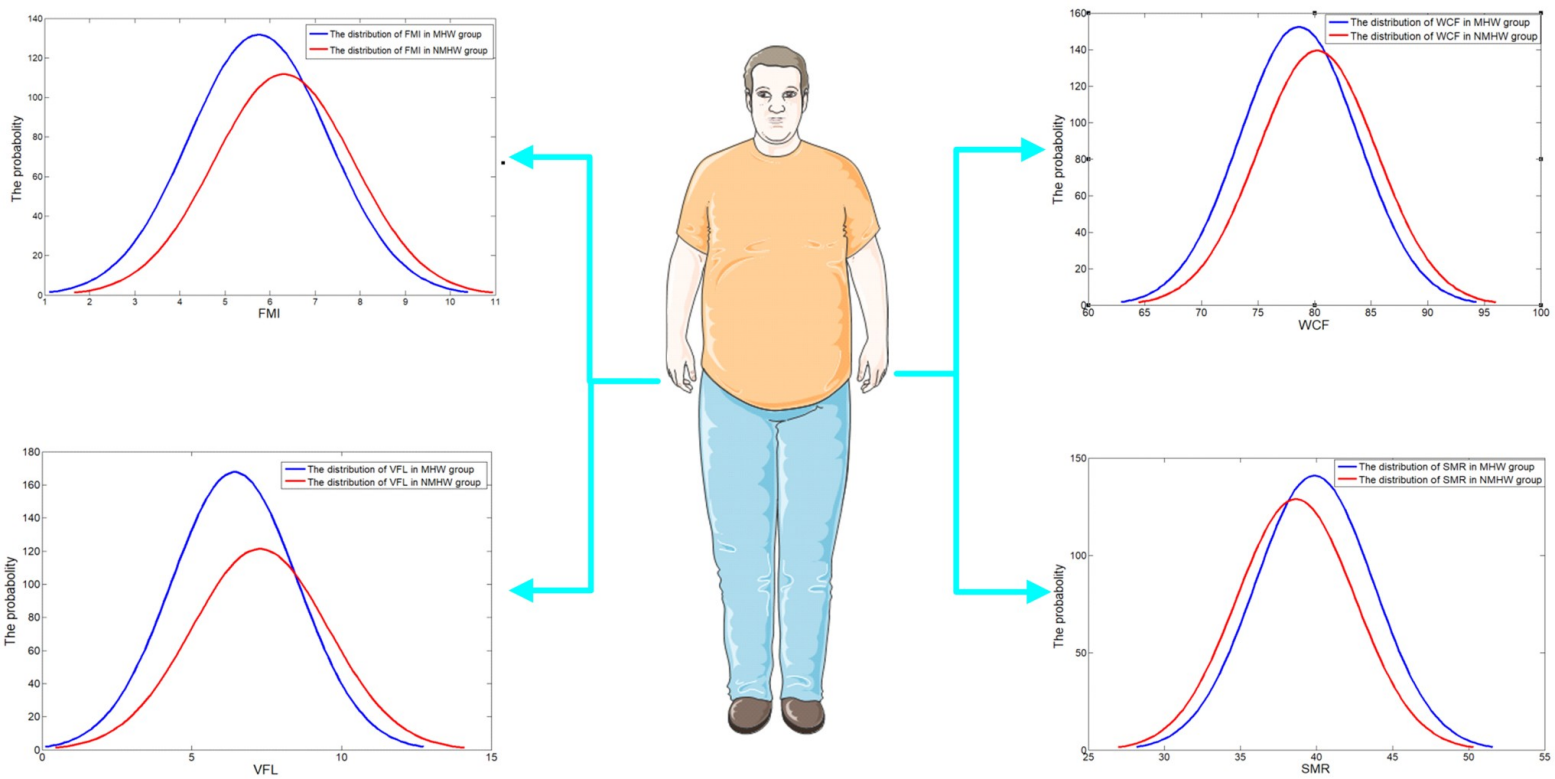
The distribution of body compositions based on the bio-electrical impedance measurements.

To examine whether the BC was independent of age and sex, we further investigated the characteristics of BC among young adult (35–44 years old), the middle aged (45–59 years old) and the older (60–74 years old) groups stratified by sex. T-tests in Table 2 showed that the FMI, VFL and WCF between MUHNW and MHNW groups were significantly different stratified by sex except SMR in aged 35–44 years group. Moreover, among the MUHNW and MHNW groups respectively, the VFL and FMI were significantly higher in individuals aged 60–74 years than aged 35–45 years and 45–59 years, independent of sex (P < 0.05). The SMR and WCF in individuals aged 60–74 years were significantly lower than the others groups (P < 0.05). The distribution of BCs based on the bio-electrical impedance measurements results are shown in Fig 2.

**Table 2:** Characteristics of body measurements of the subjects in different metabolic status and age.

Body Measurements	35–44 years old group		*t*	45–59 years old group		*t*	60–74 years old group		*t*	F^1^ (MHNW)	F^2^ (MUHNW)
	MHNW (121M/395F)	MUHNW (42M/139F)		MHNW (548M/1024F)	MUHNW (235M/554F)		MHNW (833M/560F)	MUHNW (388M/588F)			
n	516	181		1572	789		1393	976			
FMI											
Male	4.41 ± 1.18	4.81 ± 1.17	1.92	4.63 ± 1.21	5.11 ± 1.12	5.10*	4.90 ± 1.25	5.33 ± 1.38	5.46*	13.36*	4.62*
Female	6.13 ± 1.23	6.40 ± 1.32	2.22*	6.50 ± 1.25	6.72±1.29	3.43*	6.80 ± 1.44	7.12 ± 1.39	3.88*	30.92*	22.13*
VFL											
Male	4.96 ± 1.60	5.50 ± 1.67	1.86	5.30 ± 1.62	6.02 ± 1.65	5.63*	5.68 ± 1.69	6.36 ± 1.95	5.89*	15.37*	5.73*
Female	6.55 ± 1.86	7.18 ± 2.06	3.34*	7.09 ± 1.96	7.59 ± 2.11	4.64*	7.69 ± 2.41	8.24 ± 2.41	3.89*	35.68*	18.79*
WCF											
Male	80.60 ± 5.14	82.77 ± 4.45	2.43*	80.13 ± 4.96	82.86 ± 5.08	7.01*	78.29 ± 5.79	80.47 ± 5.43	6.24*	23.49*	16.56*
Female	78.75 ± 4.47	80.86 ± 5.24	4.24*	78.74 ± 4.76	80.28 ± 4.68	6.14*	76.93 ± 5.40	78.67 ± 5.26	5.53*	27.66*	19.57*
SMR											
Male	44.27 ± 2.75	43.58 ± 2.86	1.38	43.57 ± 2.84	42.68 ± 2.62	4.10*	42.18 ± 2.86	41.41 ± 3.19	4.21*	56.03*	19.75*
Female	38.79 ± 2.62	38.46 ± 2.66	1.26	37.83 ± 2.67	37.52 ± 2.85	2.14*	36.33 ± 2.96	35.97 ± 3.03	2.08*	99.85*	62.46*

*P < 0.05. Data are expressed as mean ± SD. *P*-values were calculated by an independent two-sample t-test. F1 and F2 were calculated by analysis of variance (ANOVA) within MHNW and MUHNW groups across the age groups, respectively.

### 3.3 The percentage of MUHNW varied with body compositions and age

**Fig 3:**
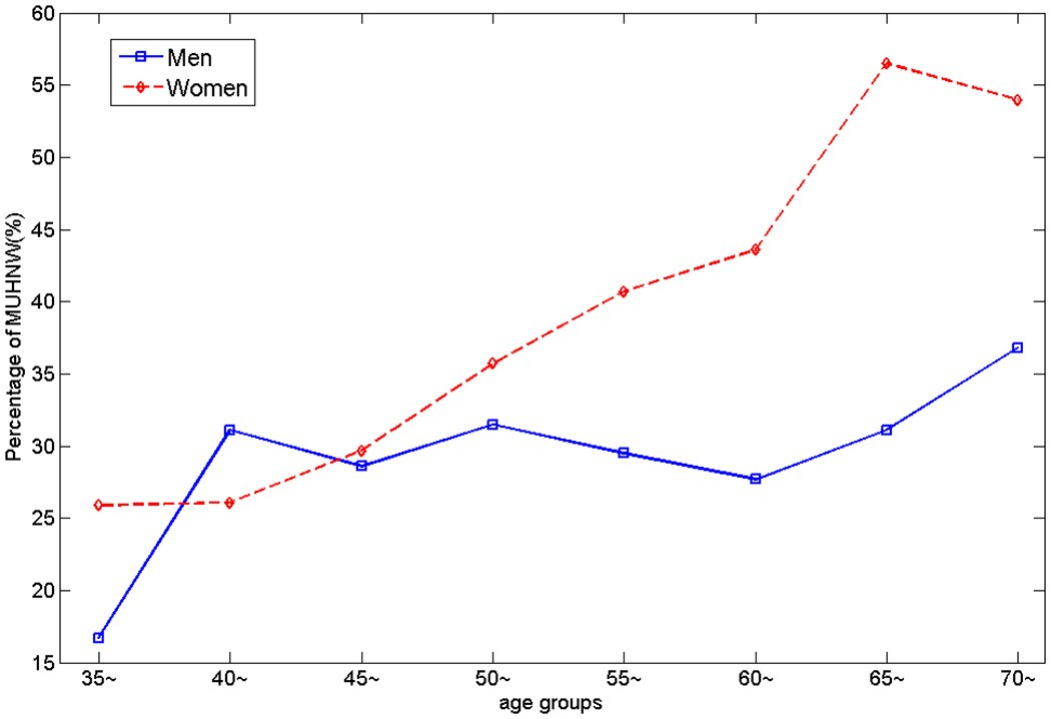
The MUHNW percentage between male and female for different age groups.

In summary, the distribution of MUHNW percentage between male and female varied by age. The percentage of the MUHNW profile significantly increased in women after the age of 55, contrary to men. There were no significant differences found for the aged 35–54 years groups between male and female (Fig 3).

**Fig 4:**
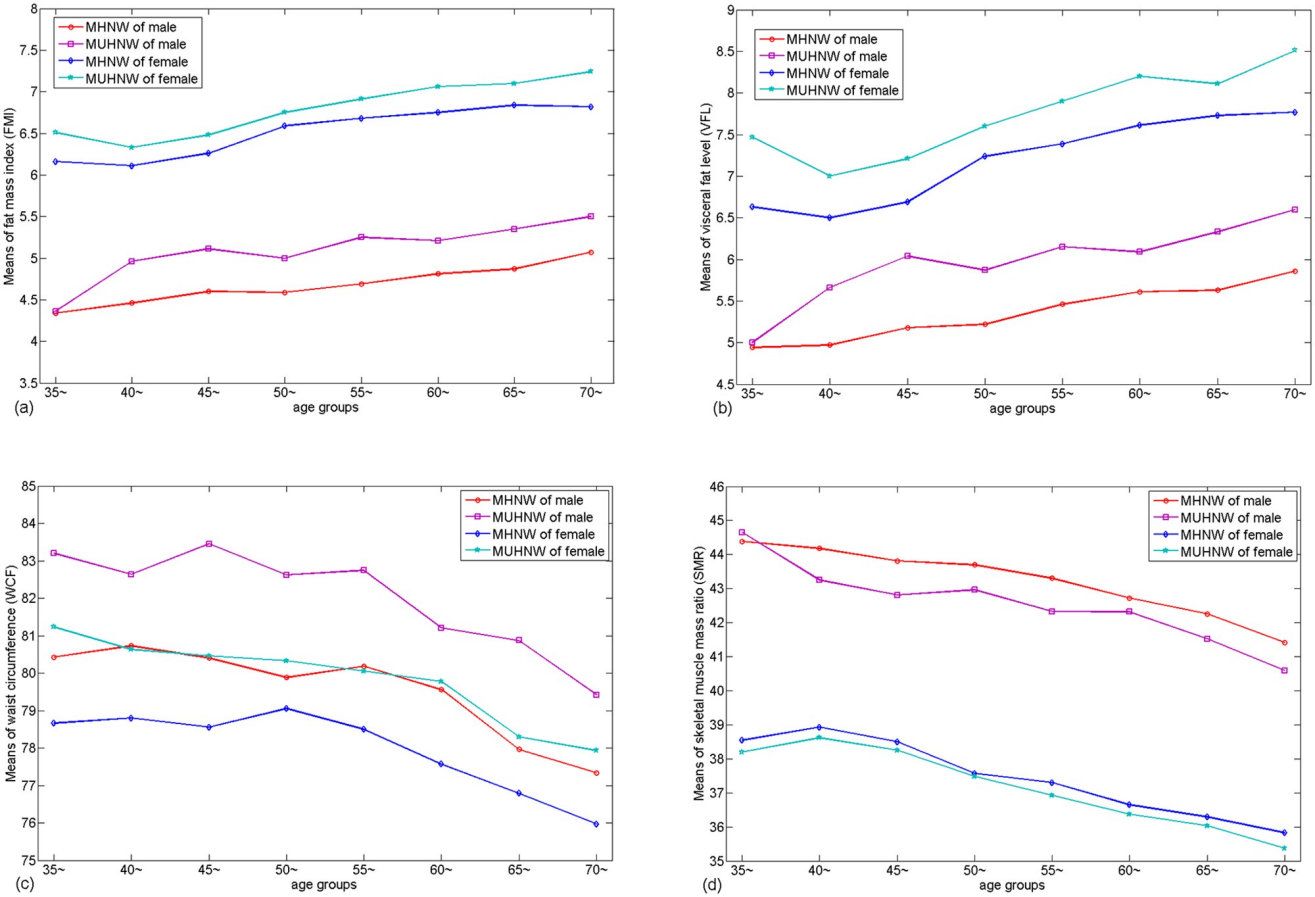
The means of FMI, VFL, WCF and SMR in different age groups.

To explore the difference of FMI, VFL, WCF and SMR between the MHNW and MUHNW with age varied, we compared the BC indices FMI, VFL, WCF and SMR respectively. More precisely, Fig 4 demonstrated that increased of FMI and VFL with age increase. By contrast, the decreased of WCF and SMR with age increase. The FMI, VFL and WCF were higher (the SMR was lower) in MUHNW than that in MHNW and they were statistically significant in most age groups.

### 3.4 Association between BC and MUHNW

**Fig 5:**
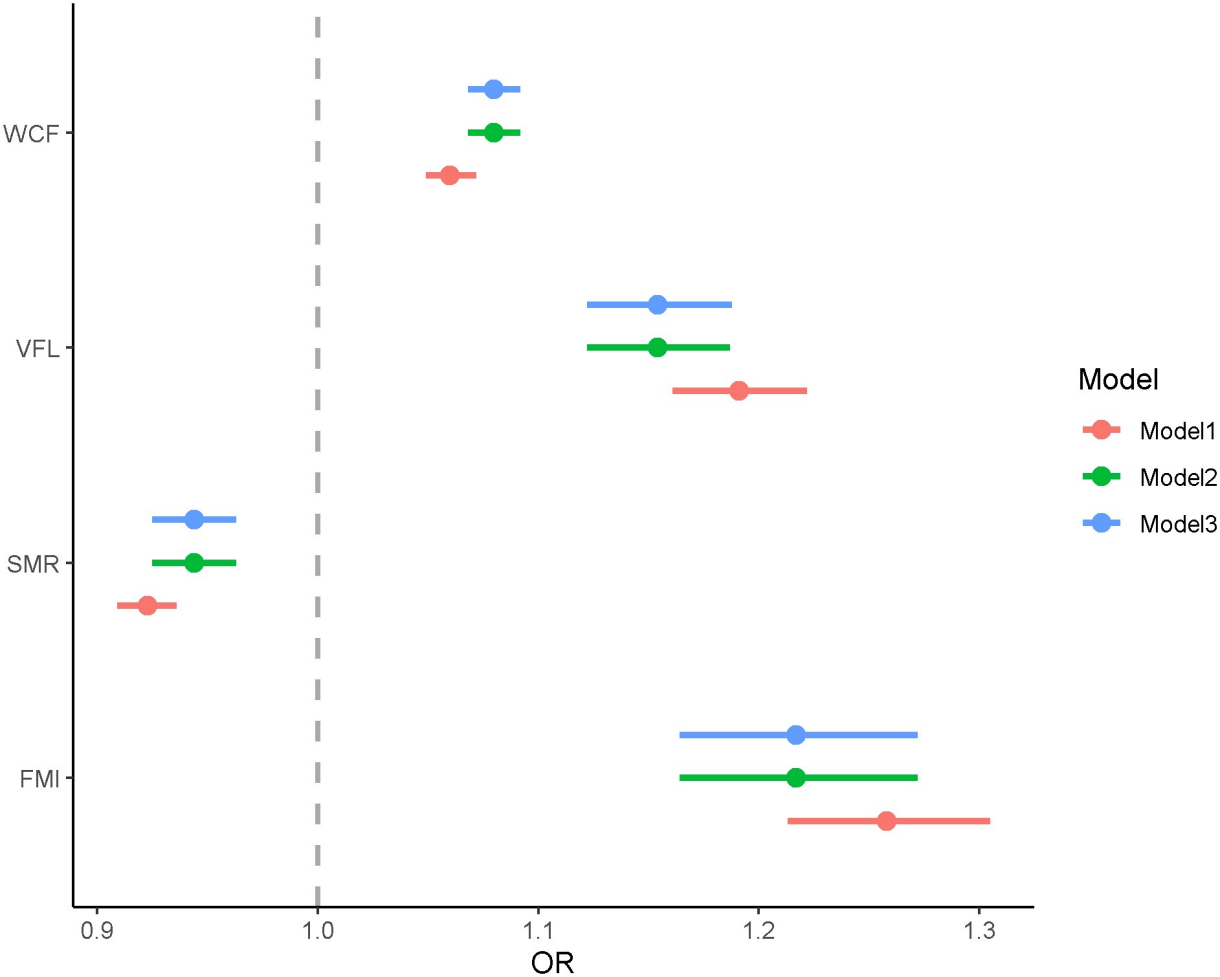
The regression results (odds ratios) between BC and MUHNW based on adjusted models.

By using GLM, we examined the associations between BC and metabolically unhealthy profile in normal weight population. As shown in Table 3 and Fig 5, the FMI, VFL and WCF were the risk factors for MUHNW, the OR (95% CI) of FMI was 1.22 (1.16, 1.27), VFL was 1.15 (1.12, 1.19), and WCF was 1.08 (1.07, 1.09) respectively. These results implied that an unit increase in FMI, may increase the risk of MUHNW by 21.7%, an unit increase of VFL may increase the risk of MUHNW 15.4%, and an unit increase of WCF may increase the risk of MUHNW by 8%. Thus, FMI, VFL and WCF were more likely to be risk factors of MUHNW, serving as an indicator of early prevention of metabolically unhealthy profile in the population with normal weight.

**Table 3:** The GLM result between body compositions and metabolically unhealthy profile.

Variables	Model 1	Model 2	Model 3
	OR (95%CI)	OR (95%CI)	OR (95%CI)
FMI	1.26(1.21,1.31)	1.22(1.16,1.27)	1.22(1.16,1.27)
VFL	1.19(1.16,1.22)	1.15(1.12,1.19)	1.15(1.12,1.19)
WCF	1.06(1.05,1.07)	1.08(1.07,1.09)	1.08(1.07, 1.09)
SMR	0.92(0.91,0.94)	0.94(0.93,0.96)	0.94(0.93,0.96)

Model 1: rude model.

Model 2: adjusted for age and sex.

Model 3: model 2+smoking, drinking, tea, exercise, sleep, and education.

Moreover, higher SMR were negatively associated with an increased MUHNW risk, and the OR (95% CI) of SMR was 0.94 (0.93, 0.96) with robustness. An unit increase of SMR may decrease the risk of MUHNW by 5.6%. Therefore, SMR may be a protective factor for the metabolically unhealthy in individuals with normal weight.

## 4. Discussion

The individuals with obesity have received enough attention, considering their apparent serious metabolic syndrome consequences, whereas there are no standardized criteria to classify the metabolically unhealthy profile of the adults with normal weight. In fact, because of the undetected individuals with MUHNW, they will more likely suffer from hyperinsulinemia, insulin-resistance, and predisposed type 2 diabetes, hypertriglyceridemia, and premature coronary heart disease since they lack enough early attention and prevention [5]. It is therefore urgent to establish some novel effective indices to early identify MUHNW from the adults with normal weight.

Some studies found that BC measured by BIA method showed high accuracy and excellent correlation with that by dual-energy X-ray absorptiometry (DXA) in assessing fat mass (FM) and skeletal muscle mass (SM) [23]. Eickemberg et *al*. [24] found that BIA was found to have satisfactory sensitivity and specificity to predict visceral fat. Thus, BIA has been considered as the simplest, most reproducible, and least expensive method for BC evaluation.

In this study, we use the baseline data of CNC-NX Cohort to examine the association between BC and metabolically unhealthy profile of adults with normal weight in Northwest China. We found that there are 35.86% of the adults with normal weight with metabolically unhealthy profile. Our findings showed that MUHNW distribution between male and female varies with age, and most ages prove to be positively correlated with the percentage of MUHNW in females. The association between BC and MUHNW may change with age and sex. The increased adiposity indices (FMI, VFL and WCF), and reduced SMR could explain the difference between MUHNW and MHNW. Wang et *al*. estimated [25] the prevalence of MUHNW in adults varied from 26% to 36% by meta-analysis, consistent with our result, but slightly higher than the average prevalence of MUHNW (30%). This variation may be due to individual characteristics such as age, sex, smoking, drinking, exercise, education, sleep situation and MUHNW definitions [26].

By using generalized linear model, we examined the associations between BC and metabolically unhealthy profile in individuals with normal weight. Our results suggested that FMI, VFL and WCR were the risk factors of increased MUHNW risk, and SMR was a protective factor of increased MUHNW risk (see Table 3). These findings indicated that the total fat, central obesity, and adipose tissue located around the internal organs may be closely associated with metabolically unhealthy profile of adults with normal weight in China.

In fact, increased visceral fat mass is associated with ectopic lipid deposition in metabolically important organs (the liver, the skeletal muscle, and the pancreas), and also contributes to the pathophysiology of cardiometabolic disease via dysregulated adipokine secretion, subclinical inflammation, and increased release of fatty acids into the circulation [27]. Existed results have shown a significant association between VAT and metabolic diseases [12]. Lu et *al*. [28] identified excess abdominal visceral fat accumulation as a major characteristic of MUHNW, with it being clinically important when assessing MetS in those with normal weight. Tatsumi et *al*. [29] demonstrated a dose-dependent relationship observed between visceral fat area (VFA) and metabolic risk factors among people with normal BMI. Katsuki et *al*. [30] found that the increased visceral fat areas in the individuals with MUHNW may be a primary cause of their increased cardiometabolic risk. In addition, a cohort study by Wang et *al*. [31] found that visceral adiposity index (VAI) is the best index for the diagnosis of Mets. In addition, VFL and WCF were associated with homeostasis model assessment of insulin resistance (HOMA-IR) in young male adults in Indonesia. Liu et *al*. [32] found that higher FMI levels appear to be independently associated with the presence of Mets. Chinese individuals tend to accumulate fat intra-abdominally and thus even lean individuals may have intra-abdominal obesity [10]. Thus, FMI, VFL and WCF would be better screening tool in prediction of the presence of metabolic syndrome. Meanwhile, reducing overall body fat and visceral fat may be conducive to preventing MUHNW early.

The function of skeletal muscle is very important in maintaining optimal health throughout life. Skeletal muscle constitutes the largest insulin-sensitive tissue in the body and is the primary site for insulin-stimulated glucose utilization. Skeletal muscle resistance to insulin is fundamental to the metabolic dysregulation associated with obesity and physical inactivity, contributing to the development of the metabolic syndrome [33]. Srikanthan et *al*. [34] found that a higher muscle mass (relative to body size) is associated with better insulin sensitivity and lower risk of prediabetes or overt diabetes. In the Korean sarcopenic obesity study, they used the thigh muscle of cross-sectional area as an index of muscle mass, and found that it was lower in MUHNW individuals [35]. These studies suggest that higher muscle mass may promote metabolic health, which is consistent with our result. More specifically, normal weight, lower fat and higher muscle mass may contribute to metabolic health, as observed by Xia et *al*. [36]. They also presumed that decreased SMR accompanied by the increased fat accumulation may play a critical role in the pathological process of the metabolically unhealthy profile.

The strengths of our study include the large sample size and that the data are from a cohort design in which people are observed in their natural environment. The strict and standardized protocols of the cohort regarding the collection of data from questionnaires, anthropometric measures and blood samples should also be acknowledged. However, this study also has its limitations. First, the cross-sectional design limited our ability to infer causality for the associations observed. Second, we only focused on rural areas, and care should be taken when extending the results to urban areas. Third, as at now, no standardized criteria exist for the definition of metabolically unhealthy profile with normal weight. We adopted a strict definition of ATP III for metabolically unhealthy profile, and our results might vary with different criteria. Fourth, BIA is less accurate at detecting visceral fat. Its principle is based on the passage of the electric current in water included in the body, through the electrodes, subdividing the body in two compartments: the fat mass (FM) where the water does not circulate and the fat free mass (FFM) including all the other compartments (water, muscle mass). Finally, the lower body fat mass (LBF) is also important for predicting the incidence of cardiometabolic disease [11]. However, owing to the inaccuracy of measured LBF results based on BIA, we did not consider LBF in our analysis, which may be improved if we have a more accurate measurement based on MRI.

## 5. Conclusions

In summary, this study showed that the percentage of MUHNW in adults with normal weight is 35.86%, and the association between MUHNW and BC varied with age and sex. The increased adiposity indices (FMI, VFL and WCF), and reduced SMR could be responsible for the difference between MUHNW and MHNW. These findings can provide new insights into the potential metabolic risk for adults with normal weight in China, and are conducive to manage MUHNW for its early prevention.

## Supporting information

S1 Dataset (XLS)

S1 File (DOC)

S1 Data (XLS)

S2 Data (XLS)

S3 Data (XLS)
