# Supplementary material for: The association between body composition and metabolically unhealthy profile of adults with normal weight in Northwest China
Source: PLoS One. 2021 Mar 25;16(3):e0248782. doi: 10.1371/journal.pone.0248782 (PMC7993598; doi:10.1371/journal.pone.0248782)
Supplement: S1 File — (DOC) [file pone.0248782.s002.doc]

**S1 File. Description of Variables in the dataset**

Sex: 1=Male; 2=Female

Education level: 1=Primary school or below; 2=Secondary school; 3=College or upper

Smoking status: 1=Non-smoking; 2=Occasional smoking; 3=Smoking on most days; 4=Smoking everyday

Alcohol drinking status: 1=No; 2=Yes

Tea drinking status: 1=No; 2=Yes

Physical exercise: 1=Never or almost never; 2=< 3 times/week; 3=≥ 3 times/week

Sleep disorder: 1=No; 2=Yes

Group: 1=MHNW; 2=MUHNW

Age-2: 1=35-44 years old group; 2=45-59 years old group; 3=60-74 years old group

Age-3:1=35-39 years old group; 2=40-44 years old group; 3=45-49 years old group; 4=50-54 years old group; 5=55-59 years old group; 6=60-64 years old group; 7=65-69 years old group; 8=70-74 years old group.
